# A case-crossover analysis to quantify the impact of wildfire smoke on hospital respiratory admissions in the Rogue Valley, Oregon^☆^

**DOI:** 10.1016/j.puhip.2024.100540

**Published:** 2024-08-23

**Authors:** A Lee Mitchell, Kyle Chapman, Kerry Farris, Pooya Naderi, Ashley Hansen

**Affiliations:** a Oregon Institute of Technology AIRE Center, Klamath Falls, OR, USA; b Oregon Institute of Technology, Humanities and Social Sciences Department, AIRE Center, Klamath Falls, OR, USA; c Oregon Institute of Technology, Natural Sciences Department, AIRE Center, Klamath Falls, OR, USA

**Keywords:** Rogue valley, Oregon, Wildfires, PM_2.5_, Air quality, Respiratory health, Hospital planning

## Abstract

**Background:**

With the increasing prevalence of wildfire smoke in the Pacific Northwest, it is important to quantify health impacts to plan for adequate health services. The Rogue Valley region has historically faced some of the greatest wildfire threats in the state. Health impacts from smoke have been estimated in several recent studies that include Oregon's Rogue Valley, but the results between studies are conflicting.

**Objective:**

The objective is to critically examine impacts of wildfire smoke on health in the Rogue Valley area and translate the results to support hospital staffing decisions.

**Study design:**

The study adopts a case-crossover approach.

**Methods:**

Apply a conditional Poisson regression to analyze time stratified counts while controlling for mean temperature.

**Results:**

Every 10 μ/m^3^ increase in PM_2.5_ is associated with a 2% increase in same-day hospital or emergency room admission rates for respiratory conditions during fire season after adjusting for temperature and time (OR = 1.020; 95% CI: 1.004–1.034); a 10 μ/m^3^ increase in PM_2.5_ lasting nine days is associated with a 4% increase in admission rates (OR = 1.041; 95% CI: 1.018–1.065). In other words, for each 10 μ/m^3^ single day increase in pollution from smoke, an additional 0.26 respiratory patients would be expected in the area hospitals. With a single day increase from 10 μ/m^3^ to 150 μ/m^3^, hospitals could expect an additional four patients.

**Conclusions:**

There are small but significant health impacts in the Rogue Valley. These impacts are smaller than some statewide estimates. We need further research to understand these differences.

## 1 What this study adds

• Contributes to evidence of wildfire smoke health impacts in a region with contradictory findings previously reported.

## 2 Implications for policy and practice

• Analysis results of health impacts from wildfire smoke are translated into hospital burden estimates to facilitate healthcare planning.

## 3 Introduction

Climate change is increasing the risk of wildfires worldwide. Wildfires and associated fine particulate matter (PM_2.5_) pollution from the smoke are a growing problem across the western US [1,2]. As a result, more people will be exposed to hazardous levels of wildfire smoke and experience the negative health effects of particulate matter pollution [3].

Elevated levels of PM_2.5_ increase the rate of people seeking medical treatment for asthma [[4], [5], [6], [7], [8], [9]]. While the intensity of effect varies among studies, a meta-analysis found that a 10 μ/m^3^ increase in PM_2.5_ was associated with risk ratio of 1.07 (95% CI: 1.04–1.09) for asthma-related emergency department visits [10]. Impacts from smoke exposure can be influenced by a variety of factors, from the composition of material fueling the fire to the behavior of people in the area [4].

**Fig. 1:**
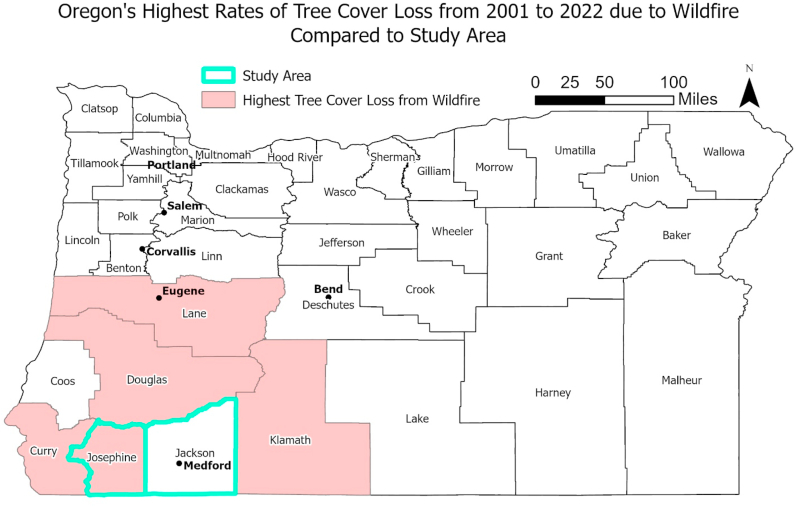
Five counties in Oregon with the highest rates of tree cover loss from 2001 to 2022 due to wildfire compared to the location of the study area.

The study area, shown in Fig. 1, covers the Rogue Valley area and suffers some of the highest rates of tree loss due to wildfires within the Oregon [11]. Recent studies on respiratory health impacts from wildfire smoke in the region present conflicting results. Gan et al. [12] found significant increases in respiratory health care utilization related to wildfire smoke concentrations during the 2013 wildfire season using a case-crossover analysis; yet Powell and Boyd [13] did not find any significant increases in asthma-related hospital visits during the 2015 wildfire season using the state's syndromic surveillance system. The Gan et al. [12] study also focused on Medford, one town in the Rogue Valley region explored in the current study, but did not find a significant relationship between asthma-related emergency room visits at that scale. In contrast, Chapman et al. [14] documented a significant risk of Rogue Valley hospitals exceeding patient capacity during the 2018 wildfire season when PM_2.5_ concentrations substantially elevated. The current study further explores the risk of adverse health outcomes in the Rogue Valley region by expanding the timeframe under study.

The aim of the current research is to model the effects of wildfire generated PM_2.5_ concentrations on respiratory hospital admission rates and present the results in a format suitable for supporting hospital staffing and resource allocation decisions. Results rely on a case-crossover analysis to estimate the impact of PM_2.5_ on respiratory hospitalizations from 2016 to 2019 in the Rogue Valley area, encompassing Josephine and Jackson Counties and the cities of Grants Pass, Medford, and Ashland.

## 4 Methods

Time-stratified case-crossover analysis designs are widely used for analyzing the health impacts of air pollution [15]. The design is suitable when the pollution exposure varies within a short timeframe, the onset of disease is abrupt, and there is a short timeframe between exposure and disease onset [16]. The population for a case-crossover analysis serves as their own controls with reference periods that can be established based on exposure characteristics [17]. Such a time-stratified design evaluates risk from exposure across time rather than between individuals, thus mitigating the risk of overlap bias [15]. The reference periods allow for controlling fixed and time-varying covariates like seasonal and secular trends, such as wintertime cold and flu season and workweek versus weekend behavioral differences [16,[18], [19], [20]]. Advantages over using Poisson time-series regressions include the ability to control for temporal confounding by design and fewer arbitrary decisions such as choosing specific covariates or the type of smoother [15,21,18]. Case-crossover designs are flexible and can employ various types of regression models in the analysis. A conditional Poisson model can avoid overdispersion and autocorrelation issues and is appropriate for community level exposure data [22,23].

This study relied on a case-crossover analysis using conditional Poisson regression for analysis of time stratified counts while controlling for mean temperature to explore the impact of wildfire smoke on health outcomes in the Rogue Valley area of Oregon. The analysis is focused on the 2016–2019 fire seasons, from June through September. This mitigates the influence of seasonal trends unrelated to wildfire smoke exposure and allows for comparing results directly to existing research. Air quality and temperature data were analyzed in relation to daily patient counts.

This secondary analysis relies on de-identified patient data as the dependent variable and PM_2.5_ concentrations as the independent variable while controlling for temperature and time. The Southern Oregon Institutional Review Board determined this research was exempt from IRB oversight on May 1, 2019. The software used in this analysis included: Microsoft's Access and Excel for data management and descriptive statistics; ESRI's ArcGIS Pro 3.1.0 for geoprocessing; and RStudio version 2023.06.0 with the gnm, stats, and tsModel libraries for the case-crossover analysis. The R code, derived from Armstrong, Gasparrini and Tobias [22], is in the supplemental materials.

### 4.1 Data

**Fig. 2:**
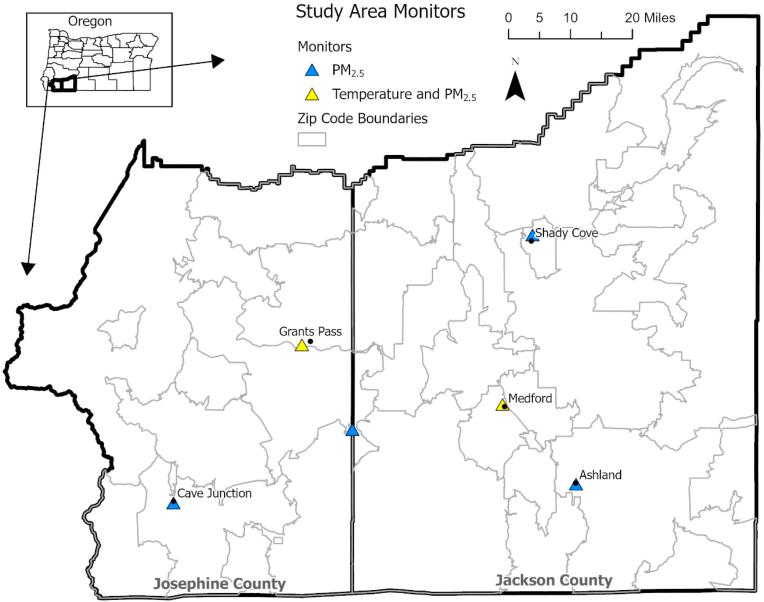
Map of study area with monitor locations and zip code boundaries in Josephine and Jackson County, Oregon.

Six air quality monitors, shown in Fig. 2, provided PM_2.5_ concentration data. The data were downloaded using Oregon Air Quality Monitoring reports available through https://oraqi.deq.state.or.us [24]. Mean daily averages were divided by 10 to provide the customary 10 μ/m^3^ reporting metrics. Missing data was imputed as the average of values from the reporting set of monitors, impacting 93 records of the 1464 total possible records, or about 6% of the data, across three of the monitors and all four years of the study time frame.

**Fig. 3:**
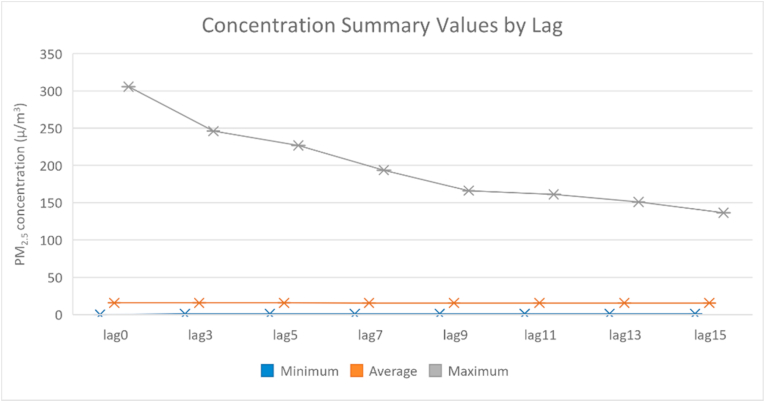
Minimum, average, and maximum PM_2.5_ concentrations per lag interval.

Exposure may not manifest as a health concern immediately, so the lag duration can impact the significance of results [14,25]. Cumulative lag values for 3-, 5-, 7-, 9-, 11-, 13- and 15-day exposures were calculated as the average concentration of the lag period from the date backward in time. Average concentrations across lag periods are shown in Fig. 3. The concentration values began to level off after nine days.

Temperature data were obtained from two meteorological monitors in the study area. Daily average temperatures were downloaded from Oregon Air Quality Monitoring reports available through https://oraqi.deq.state.or.us [24]. Both monitors were missing approximately half the data for the study timeframe; of the missing data, all but thirty records for these monitors were retrieved from the Environmental Protection Agency's Airnow site [26]. The remaining missing data was imputed by averaging temperatures from the days before and after any data gaps. Temperature values were similar across the study area, with the average minimum, mean, and maximum values within one degree between monitors, see chart in the Supplemental Materials.

The health outcome data was provided directly to the research team from hospital administration for the three out of four hospitals in the study area that use the same data management system. The data consisted of an anonymized list of all patients of any age during the 2016–2019 fire seasons that 1) went to the emergency room or were admitted to a hospital and 2) were diagnosed with a respiratory condition within the J43, J44, and J45 ICD-10 codes for emphysema, chronic obstructive pulmonary disease, and asthma, respectively. These disorders were suggested as the outcomes of interest by local hospital staff. Patient home locations were identified by zip code. Only patients with a home in one of the 25 zip codes in the study area that could be associated with the local exposure data were retained, yielding a final patient count of N = 1690.

### 4.2 Data preparation

Air quality and meteorological monitors nearest the centroid of the zip codes were spatially joined to the patient data using ArcGIS Pro. The patient data was joined to the air quality and temperature monitor data based on date, zip code, nearest air quality monitor ID, and nearest meteorological monitor ID using Microsoft Access. The data were summarized to provide daily counts of patients per zip code with the same PM_2.5_ and temperature exposures. This resulted in a final N = 12,200 (122 days * 4 years * 25 zip codes).

A year-month-day of week time stratum variable was generated for each day in Excel. This variable allows for selecting control periods on the same day of the week in the same calendar month and year. This creates a case-event day to define the case period, for example all Tuesdays in July during 2018 [15].

### 4.3 Model development

A case-crossover analysis is appropriate for exploring the impact of transitory smoke exposure on acute respiratory outcomes [15]. The patient data was provided by zip code so we were limited to community level exposure estimates, requiring an aggregated exposure case-crossover based on a Poisson regression as opposed to an individual exposure study based on conditional logistic regression [23]; though results based on a Poisson regression with an indicator variable for time strata are equivalent to a time-stratified conditional logistic design [27]. Model fit was determined by minimizing the Akaike Information Criterion (AIC) [28]. Results are considered statistically significant if the 95% confidence interval (95% CI) did not contain an odds ratio (OR) of one.

The model was developed following recommendations from Carracedo-Martínez et al. [15]. The model was first explored using baseline data without including the pollutant; the pollutant was then added to the model to identify any changes in the results. We checked the model assumptions, including independence of observations and a lack of overdispersion and outliers. Results are compared to previously published analyses to ensure reasonableness and interpreted to provide local healthcare administrators with actionable decision points regarding the impact of wildfire smoke on human health in the Rogue Valley area.

## 5 Results

The case-crossover analysis used a Poisson regression with a Brumbeck autocorrelation adjustment. Results show a 10 μ/m^3^ increase in PM_2.5_ was associated with a 2% increase in same-day hospital or emergency room admission rates for respiratory conditions during fire season after adjusting for temperature and time. Based on the population of the region, these results correspond with the expectation of an additional 0.26 respiratory patients in area hospitals for each 10 μ/m^3^ single day increase in pollution from smoke during wildfire season.

### 5.1 Exploratory statistics

The patient count during fire season is slightly but significantly correlated with the PM_2.5_ concentration (ρ = 0.04, p-value <0.0001) and negatively correlated with temperature (ρ = −0.03, p-value = 0.0034) based on the Pearson correlation coefficient. The correlation between temperature and PM_2.5_ concentrations is stronger (ρ = 0.18, p-value <0.0001).

**Fig. 4:**
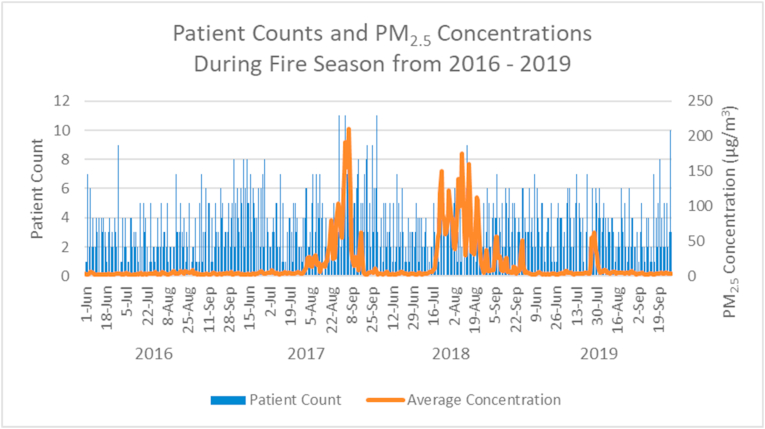
Patient count and daily average PM_2.5_ concentrations during fire season from 2016 through 2019.

Fig. 4 shows patient counts and PM_2.5_ concentrations during fire season across all four years. The increase in both is most marked during August and September 2017; another notable increase is apparent during July and August 2018. The average yearly PM_2.5_ concentration during fire season across all four study years was 15.8 μ/m^3^. The most active fire years in the region were 2017 and 2018 with an average of 22.4 μ/m^3^ and 31.5 μ/m^3^, respectively; while the average in 2016 was 3.4 μ/m^3^ and in 2019 was 6.0 μ/m^3^.

### 5.2 Case-crossover model

A conditional Poisson model was implemented in R using the gnm library with the eliminate option to analyze the time stratified counts in a case-crossover formulation. The model was first explored using baseline data without including the pollutant. When modeling just temperature on patient counts, the model had an AIC of 10501 and the temperature coefficient was not significant (95% CI OR: 0.9832–1.0022). The pollutant was then added to the model to identify any changes in the results. Including PM_2.5_ concentrations brought the AIC down to 10497.

The data were checked for outliers, independence of observations, and overdispersion to adhere to model assumptions. Diagnostic graphics are included in the Supplemental Materials. Outliers can excessively influence the model fit [27]. None of the data points fall outside of Cook's distance in the Residuals vs Leverage plot, indicating no overly influential data points. The interquartile summary of each numeric data field also demonstrated values were in expected ranges with no obvious outliers.

The case-crossover method assumes observations are independent both within and across strata. This assumption is violated if counts have residual autocorrelation [22]. For the data in this study, the Autocorrelation Function plot shows a gradual geometrically decreasing pattern, and the Partial Autocorrelation Function plot shows one significant lag followed by a drop. This indicates the data has a first order correlation [29]. The Brumback method was applied as recommended to allow for this first order correlation [22,30]. This adjustment brought the model AIC down to 10138.

Overdispersion of the data would violate assumptions of the Poisson regression approach and require use of quasi-Poisson regression. The residual deviance of the model is 6918.6 on 12084 degrees of freedom. Because the residual deviance is less than the degrees of freedom, the overdispersion assumption has not been violated so a Poisson regression is appropriate [22].

**Fig. 5:**
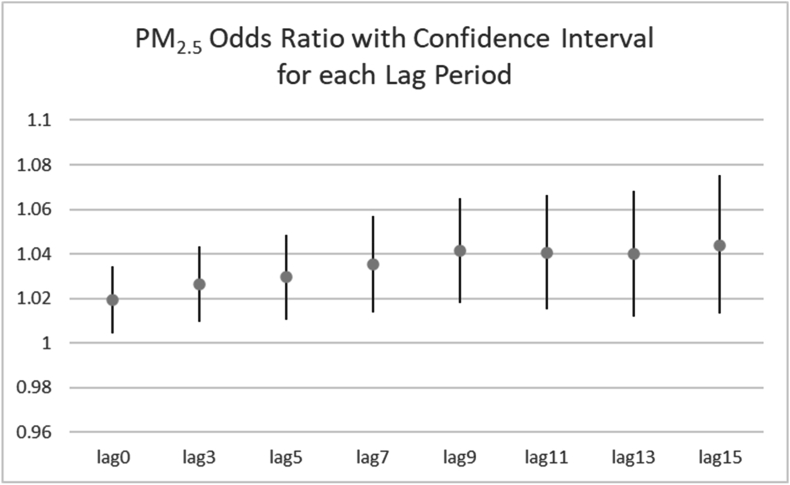
PM_2.5_ odds ratios with confidence intervals for each lag period.

PM_2.5_ concentrations were significant in the final model. Every 10 μ/m^3^ increase in PM_2.5_ is associated with a 2% increase in same-day hospital or emergency room admission rates for respiratory conditions during fire season after adjusting for temperature and time (OR = 1.020; 95% CI: 1.004–1.034). This impact increases along with an increasing duration of smoke as measured by the cumulative lag periods described in section 2.1. The impact levels out after about a week of increased smoke concentrations, as shown in Fig. 5. The nine-day leveling period is consistent with the levelling of pollution concentrations during the study timeframe after nine days, shown in Fig. 3. A 10 μ/m^3^ increase in PM_2.5_ lasting over a nine-day lag period is associated with a 4% increase in respiratory admission rates (OR = 1.041; 95% CI: 1.018–1.065).

The baseline patient count for the study hospitals, N = 1,561, was determined by the average number of respiratory patients during fire season. This corresponds to an average of 13 respiratory patients per day. Each day that has a 10 μ/m^3^ increase in PM_2.5_ is associated with a 2% increase in respiratory patients. This corresponds with an additional 0.26 patients per day per 10 μ/m^3^ increase in PM_2.5_. If the PM_2.5_ concentration rises from, for example, 10 μ/m^3^ in the “good” category of the air quality index to 150 μ/m^3^ in the “unhealthy” category, this represents a fourteen-fold increase. Area hospitals should then expect an additional four patients. The total number of licensed beds at the smallest of the study hospitals is 49. If the smoke concentrations were focused around this hospital, four additional patients would represent almost 10% of the licensed beds. When the 10 μ/m^3^ increase lasts for nine days, area hospitals should expect an additional 0.51 patients per day. If the air quality index were to remain near 150 μ/m^3^ for nine days, area hospitals should expect an additional seven patients.

Lower temperatures were not significantly associated with increased patient counts during fire season (OR = 0.99; 95% CI: 0.98–1.00). This impact of temperature is consistent across the lag periods. With the study timeframe limited to summer months, the temperature minimum, maximum, and average values were almost indistinguishable between monitors as shown in the Supplemental Materials.

## 6 Discussion

Comparing the results to studies using different data and methodologies can highlight differences in the sensitivity of the results. Results did not agree with the results from Powell and Boyd [13] or the Medford-focused results from Gan et al. [12] that found no association between smoke pollution and respiratory health outcomes. The results did agree with the Gan et al. [12] statewide analysis and the Chapman et al. [14] analysis that found statistically significant relationships between smoke pollution and respiratory health outcomes.

Powell and Boyd [13] used the state syndromic surveillance system to explore health outcomes from wildfire smoke. The study encompassed two counties, one of which, Jackson County, was also included in the current study. They did not identify any marked increases in asthma respiratory emergency department visits across the state during the 2015 wildfire season. Discrepancies in results may be related to differences in the study areas. This was the first year that all eligible hospitals reported into the system. Data entry or sampling errors related to the eligibility of the hospitals to report into the system may have contributed to the discrepancy in results between the studies. Results from this study were published as a conference abstract so were not peer reviewed, limiting the reliability of the comparison.

In Gan et al. [12], across the state of Oregon a 10 μ/m^3^ increase in PM_2.5_ was significantly associated asthma diagnoses in emergency room admissions (OR: 1.089, 95% CI: 1.043–1.136) and hospital admissions (OR: 1.072, 95% CI: 0.995–1.154) for patients with a primary residence in Oregon during May through September of 2013 based on a conditional logistic regression. These impact estimates are higher than results from the current study focused on the Rogue Valley area which found a 2%–4% increase. The difference in results may indicate differences in behaviors related to smoke among regions or may indicate a higher smoke tolerance for people in the Rogue Valley. The difference may also relate to differences in smoke composition between ecosystems across the state.

Within just the town of Medford, Oregon, a time series of daily asthma rates, using a quasi-Poisson regression and a natural spline with three degrees of freedom, regressed on a binary classifier of smoke presence based on >15 μ/m^3^ PM_2.5_ concentrations in Medford between May and September 2013, found no significant associations [12]. While these findings are contradictory to those presented here, this contradiction may be related to the cutoff value of 15 μ/m^3^ PM_2.5_. If the local population has a higher tolerance for smoke due to historic exposure, the cutoff value may be too low to capture levels that would concern the local population. Differences in results may also be related to differences in the study areas. As shown in Fig. 1, Medford lies in a county with less recent historic impact to tree cover from wildfires than the surrounding areas. Smoke concentrations can also be highly variable between years. Differences in smoke exposure to the study population may help account for different results.

Chapman et al. [14] used a generalized linear model with a binomial error structure to estimate the probability of exceeding respiratory hospital burden in the Rogue Valley during 2018. Results indicate probability increases as both a function of increasing PM_2.5_ and the duration of poor air quality, with hospital admissions peaking approximately 10 days after the peak in PM_2.5_. These results are consistent with the current study, particularly regarding the effect of smoke duration increasing until after a week of higher concentrations when the impact levels off.

The current study has some limitations in terms of exposure resolution and patient data. Patient data was obtained from three of the four hospitals in the region, just under 80% of the potential hospital capacity, potentially introducing sampling bias into our results. Exposures are limited to a zip code resolution which may not reflect accurate exposures in this region. Future research should explore the impact of patient and exposure resolution in this rural area with complex terrain.

Analysis methods and data choices vary between published studies regarding health impacts from wildfire smoke. These choices can impact the results and may have impacted the comparison presented here. Future research should explore the utility of different datasets under different environmental conditions.

The Rogue Valley area of Oregon has a long history of wildfire impacts. This history may influence the behaviors people adopt during smoke events. Future research should explore behavioral adaptations in fire-prone areas that could impact health outcomes.

Delineating the health impacts of smoke exposure is crucial if we are to better prepare for wildfire events. Reliable and interpretable estimates can inform local health service providers so they can adequately prepare for wildfire events in the region.

## Declaration of competing interest

The authors declare that they have no known competing financial interests or personal relationships that could have appeared to influence the work reported in this paper.
