# Supplementary material for: A case-crossover analysis to quantify the impact of wildfire smoke on hospital respiratory admissions in the Rogue Valley, Oregon
Source: Public Health Pract (Oxf). 2024 Aug 23;8:100540. doi: 10.1016/j.puhip.2024.100540 (PMC11402308; doi:10.1016/j.puhip.2024.100540)
Supplement: Multimedia component 1 [file mmc1.docx]

**Supplemental Materials**

**R Code**

library(gnm)

library(stats)

library(tsModel)

setwd(…”/datafiles");

dt=read.csv('patcountsExps.csv')

dtfs=subset(dt, fs == '1')

dtfs$PM2510 <- dtfs$dailyAvgPM/10

dtfs$lag310 <- dtfs$lag3/10

dtfs$lag510 <- dtfs$lag5/10

dtfs$lag710 <- dtfs$lag7/10

dtfs$lag910 <- dtfs$lag9/10

dtfs$lag1110 <- dtfs$lag11/10

dtfs$lag1310 <- dtfs$lag13/10

dtfs$lag1510 <- dtfs$lag15/10

options(na.action="na.exclude")

print(acf(dtfs$PM2510,p1=TRUE))

print(pacf(dtfs$PM2510,p1=TRUE))

print(acf(dtfs$PM2510,lag=5,p1=TRUE))

# RUN THE MODEL AND OBTAIN PREDICTIONS

pmvar = "PM2510"

logcount <- log(dtfs$Count+1)

# USE FOR TESTING modelPfs <- gnm(Count ~ meanTemp, data=dtfs, family=poisson(), eliminate=factor(stratum))

modelPfs <- gnm(Count ~ get(pmvar)+meanTemp, data=dtfs, family=poisson(), eliminate=factor(stratum))

summary(modelPfs)

# ADD BRUMBACK AUTOCORRELATION ADJUSTMENT

reslagfs <- Lag(residuals(modelPfs,type="deviance"),1)

modelAdjfs <- gnm(Count ~ get(pmvar)+meanTemp + reslagfs, data=dtfs, family=poisson, eliminate=factor(stratum))

summary(modelAdjfs)

sqrt(mean(modelAdjfs$residuals^2)) #RMSE

exp(coef(modelAdjfs))

exp(cbind(OR = coef(modelAdjfs), confint(modelAdjfs))) #OR

plot(modelAdjfs) # plot observed vs predicted etc

**Additional Tables and Figures**


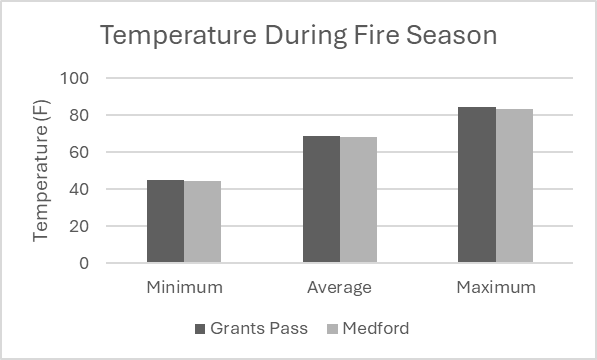


Figure 1 supplemental: Minimum, average, and maximum temperatures across the study time frame from the meteorological monitors.


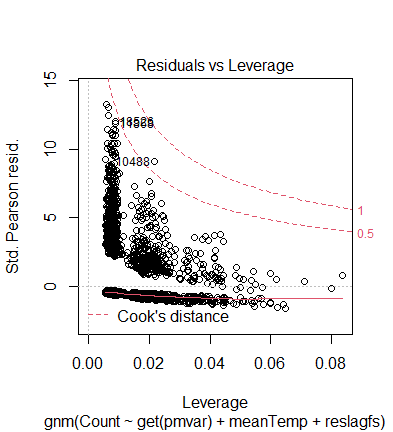


Figure 2 supplemental: Cook's distance in the residual versus leverage plot

Table 1 supplemental: Summary of interquartile values for numeric data fields.

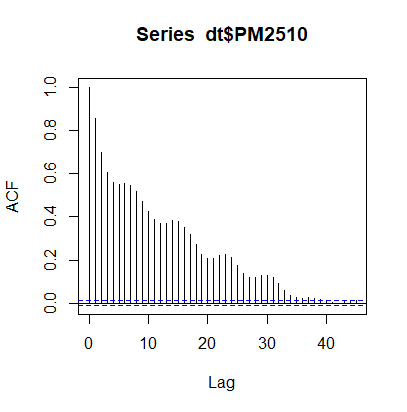


Figure 3 supplemental: Autocorrelation function plot


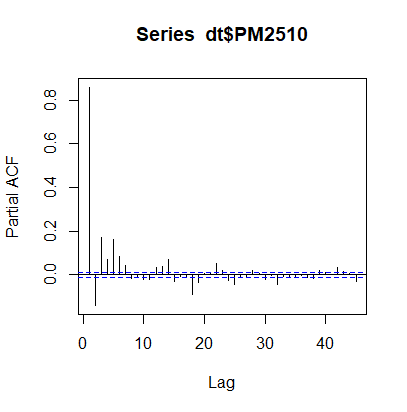


Figure 4 supplemental: Partial autocorrelation function plot
